# Antibodies to Antigenic Site A of Influenza H7 Hemagglutinin Provide Protection against H7N9 Challenge

**DOI:** 10.1371/journal.pone.0117108

**Published:** 2015-01-28

**Authors:** Falko Schmeisser, Anupama Vasudevan, Swati Verma, Wei Wang, Esmeralda Alvarado, Carol Weiss, Vajini Atukorale, Clement Meseda, Jerry P. Weir

**Affiliations:** Division of Viral Products, Center for Biologics Evaluation and Research, Food and Drug Administration, 10903 New Hampshire Ave, Silver Spring, MD 20993, United States of America; Icahn School of Medicine at Mount Sinai, UNITED STATES

## Abstract

Identifying major antigenic and protective epitopes of the H7 hemagglutinin (HA) will be important for understanding the antibody response to vaccines developed against the novel influenza H7N9 viruses that emerged in China in 2013. To facilitate antigenic characterization of the H7N9 HA and to develop reagents for evaluation of H7N9 candidate vaccines, we generated a panel of murine monoclonal antibodies (mAbs) to the HA of A/Shanghai/2/2013 using mammalian cell-derived virus-like particles (VLP) containing the H7 HA. Neutralizing antibodies identified an HA epitope corresponding to antigenic site A on the structurally similar influenza H3 hemagglutinin. Importantly, the neutralizing antibodies protect against A/Shanghai/2/2013 challenge. This antigenic site is conserved among many H7 viruses, including strains of both Eurasian and North American lineage, and the isolated neutralizing antibodies are cross-reactive with older H7 vaccine strains. The results indicate that the identified antigenic site is a potentially important protective epitope and suggest the potential benefit of cross-reactive antibody responses to vaccination with H7 candidate vaccines.

## Introduction

The novel H7N9 viruses that emerged in China in 2013 [1] resulted in severe respiratory disease in humans [2] with nearly 400 fatalities by mid-2014 (http://www.who.int/influenza/human_animal_interface/HAI_Risk_Assessment/en/). Previously reported infections with influenza viruses of the H7 subtype usually resulted in relatively mild illness in humans [3], although H7 viruses were known to occasionally infect humans with severe consequences [4, 5]. Because of the documented ability of H7 viruses to infect humans, as well as the sporadic outbreak of highly pathogenic H7 viruses in poultry, several candidate vaccine strains for the H7 subtype were developed well before the 2013 H7N9 outbreak [6–8]. Some of those earlier H7 candidate vaccine strains were evaluated in clinical trials, including an H7N7 vaccine containing the hemagglutinin (HA) from A/mallard/Netherlands/12/2000, an H7 virus of Eurasian origin that is phylogenetically related to the HA from the recent H7N9 viruses in China. Unfortunately, the immunogenicity of these earlier H7 vaccines was poor [9, 10]. More recently, candidate H7N9 vaccines have been prepared, and the results from some of the clinical trials with those vaccines have become available [11, 12]. However, at the present time, there is a relatively poor understanding of the protective immunity induced by H7 vaccines.

Identifying major antigenic and protective epitopes of the H7 hemagglutinin will be important for understanding vaccine responses. Here we report the isolation of several murine monoclonal antibodies (mAbs) that recognize the HA of the H7N9 A/Shanghai/2/2013 virus, including antibodies with neutralization and hemagglutination inhibition (HI) activity. The HA epitope recognized by the neutralizing antibodies was identified by isolation of virus escape mutants and mapped to a region analogous to the antigenic site A of influenza H3 hemagglutinin. We demonstrate that neutralizing mAbs to this site are cross-reactive to other strains of influenza H7 and are protective against an H7N9 challenge. This antigenic site is relatively well conserved among H7 virus isolates, including older vaccine strains, suggesting potential benefit of cross-reactive antibody responses to vaccination with H7 candidate vaccines.

## Materials and Methods

### Cells and viruses

Influenza viruses were propagated in 9-day-old specific pathogen-free embryonated chicken eggs. Viruses were titered by plaque assay on Madin-Darby Canine Kidney cells (MDCK) [13], originally obtained from the Centers for Disease Control. MDCK cells were used for isolation of escape mutants and were maintained in Dulbecco's Modified Eagle Medium supplemented with 10% FBS (HyClone, Logan, UT), 2 mM L-glutamine, and 50 μg/ml gentamicin.

### Monoclonal Antibodies to A/Shanghai/2/2013 HA

Monoclonal antibodies to A/Shanghai/2/2013 HA were prepared by Precision Antibody (Columbia, MD) as previously described [14]. BALB/c mice were immunized and boosted with mammalian-derived VLP [15] containing influenza A/Shanghai/2/2013 HA as the only influenza antigen.

### Passive transfer of monoclonal antibodies and animal challenge

BALBc/cByJ mice were purchased from Jackson Laboratories and housed in cages at a core facility at CBER/FDA. Sterile food and water were supplied *ad libitum*. All antibody transfers, and challenges were performed in accordance with an animal protocol approved by the Center for Biologics Evaluation and Review/FDA Animal Care and Use Committee (#2008–02); procedures were similar to those described previously [14, 15]. Monoclonal antibodies (100 μg/mouse in 0.5 ml) were delivered by intraperitoneal (i.p.) injection; for virus challenge, each mouse received 10 μl of virus suspension in the naris of each nostril (total virus – 1.4 x 10^4^ pfu) while under anesthesia (i.p. injection of Avertin (20 μl/gram body weight of an aqueous solution of tribromoethanol [17.23 grams/l]). Mice were weighed daily thereafter, and monitored for 2 weeks. Any mouse that lost 25% of body weight at any time point was sacrificed according to the approved animal protocol, by means of carbon dioxide inhalation in a euthanasia chamber where the CO_2_ obtained is from a cylinder source. Carbon dioxide is introduced at the rate of at least 20% of the chamber volume per minute and animals are observed for 10 minutes to verify death.

### Hemagglutination Inhibition and Plaque Reduction Neutralization Assays

The hemagglutination inhibition assay (HI) was performed in 96-well plates (U-bottom) by a standard method, essentially as described previously [16] using 0.5% chicken red blood cells suspended in PBS (pH 7.2). For the plaque reduction neutralization assay, virus was titrated to approximately 250 pfu/ml and incubated with concentrations of mAb from 20 to 0.032 μg/ml for 1 hour at room temperature before infection of MDCK cells. After 1.5 hour adsorption, virus inoculum was removed and the cells overlaid with a 50:50 mixture of 2X EMEM plaque media (BioWhittaker, Walkersville, MD) and 2.4% Avicel RC-581 (FMC BioPolymer, Philadelphia, PA) [17] with TPCK-trypsin (Sigma-Aldrich Corp. St. Louis, MO) at a final concentration of 2 μg/ml. At 96 hours, the plaque media was removed and the cells fixed and stained with 0.3% crystal violet/20% methanol. Percent neutralization was calculated for each mAb concentration versus no mAb controls.

### HA-Pseudotype Neutralization Assay

Retroviral pseudotypes expressing the influenza HA of A/Shanghai/2/2013 HA were constructed and were produced in 293T cells essentially as described previously [14, 18, 19]. HA-pseudotypes containing approximately 15 ng/ml of p24 antigen were incubated with antibody samples for 1 h at 37°C, then 100 μl of pseudovirus and antibody mixture was inoculated onto 96-well plates that were seeded with 2 x 10^4^ 293T cells/well one day prior to infection. The antibody dilution causing a 95% reduction of vector expressed luciferase compared to control was used as the neutralization endpoint titer (IC_95_-neutralizing antibody titer). IC_95_ was calculated using Prism software (GraphPad Software, Inc., La Jolla, CA). Data reported were from at least duplicate testing of antibody samples.

### Selection of Escape Mutants

Isolation and selection of influenza virus escape mutants to monoclonal antibodies was performed in MDCK cells. Briefly, ~2 x 10^2^ pfu of A/Shanghai/2/2013 virus was incubated with each mAb over a range of concentrations from 8.0–0.064 μg/ml (IC_50_ concentrations for 5A6, 2C4, and 4A2 were ~ 1 μg/ml) in a total volume of 1 ml for 1 hour at room temperature, followed by infection of ~ 2.5 x 10^6^ MDCK cells with the virus-antibody mixtures. After 1.5 hour adsorption, virus inoculum was removed and the cells overlaid with serum-free media containing 2 μg/ml TPCK-trypsin. Using virus obtained at the highest concentration of mAb, the process was repeated at a 5-fold higher mAb concentration for up to 3 additional rounds of neutralization. Potential escape viruses were tested for reduced inhibition of neutralization by the mAb compared to the parent virus and consensus nucleotide sequences of viral HAs were determined by direct DNA-sequencing of RT-PCR products and compared with those of the parental virus stock.

### Surface Plasmon Resonance (SPR) analysis

Surface Plasmon Resonance (SPR) analysis was performed on a Biacore T200 (GE Healthcare Bio-Sciences, Pittsburgh, PA). Antibody-antigen interactions were measured in running buffer (0.01M HEPES pH 7.4, 0.15M NaCl, 0.005% Surfactant P20) at 25°C. The surface of a CM5 sensor chip was coated with rabbit anti-mouse IgG Fcγ (RAMG) (Jackson ImmunoResearch Laboratories, Inc.,West Grove, PA) at a density of 6,000 resonance units (RU) by amine coupling technique using 1-ethyl-3-[3-dimethylaminopropyl] carbodiimide (EDC) and N-hydroxysuccinimide (Sulfo-NHS) for activation and 1M ethanolamine hydrochloride for blocking. All H7N9 monoclonal antibodies were diluted to 10 μg/ml, blocking mAb was diluted to 50 μg/ml, and the H7 HA antigen was diluted to 25 μg/ml in running buffer. One mAb was captured on the sensor chip surface via the anti-mouse IgG Fcγ by injecting the mAb at a flow rate of 5 μl/min for a contact time of 60s and then washing with running buffer for a dissociation time of 300 seconds. Free RAMG sites were blocked by injecting a non-specific mAb at 5 μl/min for 360s association and 600s dissociation. Recombinant influenza A/H7N9/Shanghai/2/2013 HA protein (Sino Biological Inc., Beijing P.R.China) was injected for a contact time of 360s association and 600s dissociation. Following the antigen each of the remaining mAbs in the test panel were injected sequentially on the sensor chip surface for a contact time of 360s association and 600s dissociation. Regeneration of the chip surface was performed with 10mM glycine-HCl pH 2.0. Binding kinetics were evaluated using Biacore T 200 evaluation software version 1.0. The procedure was repeated using each mAb in the panel as the first mAb in the binding assay. Likewise, reversed-order multi-determinant tests were performed.

For the affinity analysis, each of the mAb was captured on the sensor surface using the anti-mouse IgG Fcγ. The antigen was diluted in running buffer to a concentration of 100, 50, 25, 12.5, 6.25 and 0 nM. Each antigen concentration was injected sequentially onto the chip at a flow rate of 5 μl/min for a contact time of 60s and washed with running buffer for a dissociation time of 600s. Regeneration of the chip surface was performed with 10 mM glycine-HCl pH 2.0. Affinity analysis was performed using the single kinetic analysis method with a 1:1 binding model.

## Results

### Isolation and characterization of monoclonal antibodies to the H7 A/Shanghai/2/2013 hemagglutinin

To facilitate antigenic characterization of the H7N9 HA and to develop reagents for evaluation of H7N9 candidate vaccines, we generated a panel of murine monoclonal antibodies (mAbs) to the HA of A/Shanghai/2/2013. Mammalian-derived virus-like particles (VLP) containing the hemagglutinin (HA) of the influenza H7N9 A/Shanghai/2/2013 virus were prepared as previously described [15] and used to immunize mice and generate hybridoma clones secreting mAbs to H7 HA [14]. Antibodies were assessed for binding to the A/Shanghai/2/2013 HA in an ELISA using the inactivated candidate vaccine virus A/Shanghai/2/2013 (H7N9) IDCDC-RG32A (http://www.who.int/influenza/vaccines/virus/en/) and five mAbs with the strongest binding to A/Shanghai/2/2013 HA were selected for further characterization (Table 1). All five H7 mAbs also bound an older H7N7 vaccine virus [9] containing the A/mallard/Netherlands/12/2000 HA. The three antibodies that bound most strongly to A/Shanghai/2/2013 HA in the ELISA assay inhibited A/Shanghai/2/2013 hemagglutination more strongly than other two H7 mAbs (4F12, 6D1) when analyzed in a hemagglutination inhibition (HI) assay (Table 1).

**Table 1:** Characterization of H7 A/Shanghai/2/2013 monoclonal antibodies.

H7 mAb	A/Shanghai/2/2013 ELISA End-point Titer (μg/ml)^a^	A/mallard/NL/12/2000 ELISA End-point Titer (μg/ml)^a^	A/Shanghai/2/2013 HI Titer (μg/ml)^b^	A/Shanghai/2/2013 Pseudotype Neutralization Titer (μg/ml)^c^
5A6	3.1 x 10^-4^	1.6 x 10^-4^	6.25	<0.0625
2C4	1.6 x 10^-4^	7.8 x 10^-5^	3.125	0.144
4A2	1.6 x 10^-4^	7.8 x 10^-5^	3.125	0.161
4F12	1.3 x 10^-3^	7.8 x 10^-4^	200	>8
6D1	1.3 x 10^-3^	6.3 x 10^-2^	238	>8

^a^ The end-point titer was defined as the lowest antibody concentration that gave an absorbance value at 405 nm value that was greater than 0.05.

^b^ Lowest antibody concentration that inhibited A/Shanghai/2/2013 hemagglutination of chicken red blood cells; initial mAb concentration 400 μg/ml.

^c^ Antibody concentration resulting in a 95% reduction in relative luciferase units of retrovirus pseudotype expressing A/Shanghai/2/2013 H7 HA.

Further characterization of the neutralizing potential of the five H7 mAbs was carried out using a pseudotype neutralization assay [18, 19] (Table 1). In this assay, retroviral pseudotypes expressing influenza A/Shanghai/2/2013 HA were incubated with individual antibodies, followed by infection of 293T cells and measurement of vector expressed luciferase. Three antibodies (5A6, 2C4, and 4A2) neutralized H7 A/Shanghai/2/2013 HA-containing pseudotype viruses more strongly than the other mAbs (4F12, 6D1), consistent with the results of the A/Shanghai HI assay.

### Monoclonal antibody epitope mapping

A initial experiment using Surface Plasmon Resonance (SPR) was designed to determine whether the 3 antibodies exhibiting A/Shanghai HI and pseudotype neutralization activity competed for the same epitope. Each of the 3 neutralizing mAbs readily bound recombinant influenza A/H7N9/Shanghai/2/2013 HA protein (Sino Biological Inc.), but subsequent addition of the other two mAbs resulted in little additional binding, suggesting that 5A6, 2C4, and 4A2 epitopes overlap (data not shown). In addition, the KD values (affinity) determined by SPR analysis were in the 10^-6^ M range for all these mAbs (data not shown).

**Figure 1:**
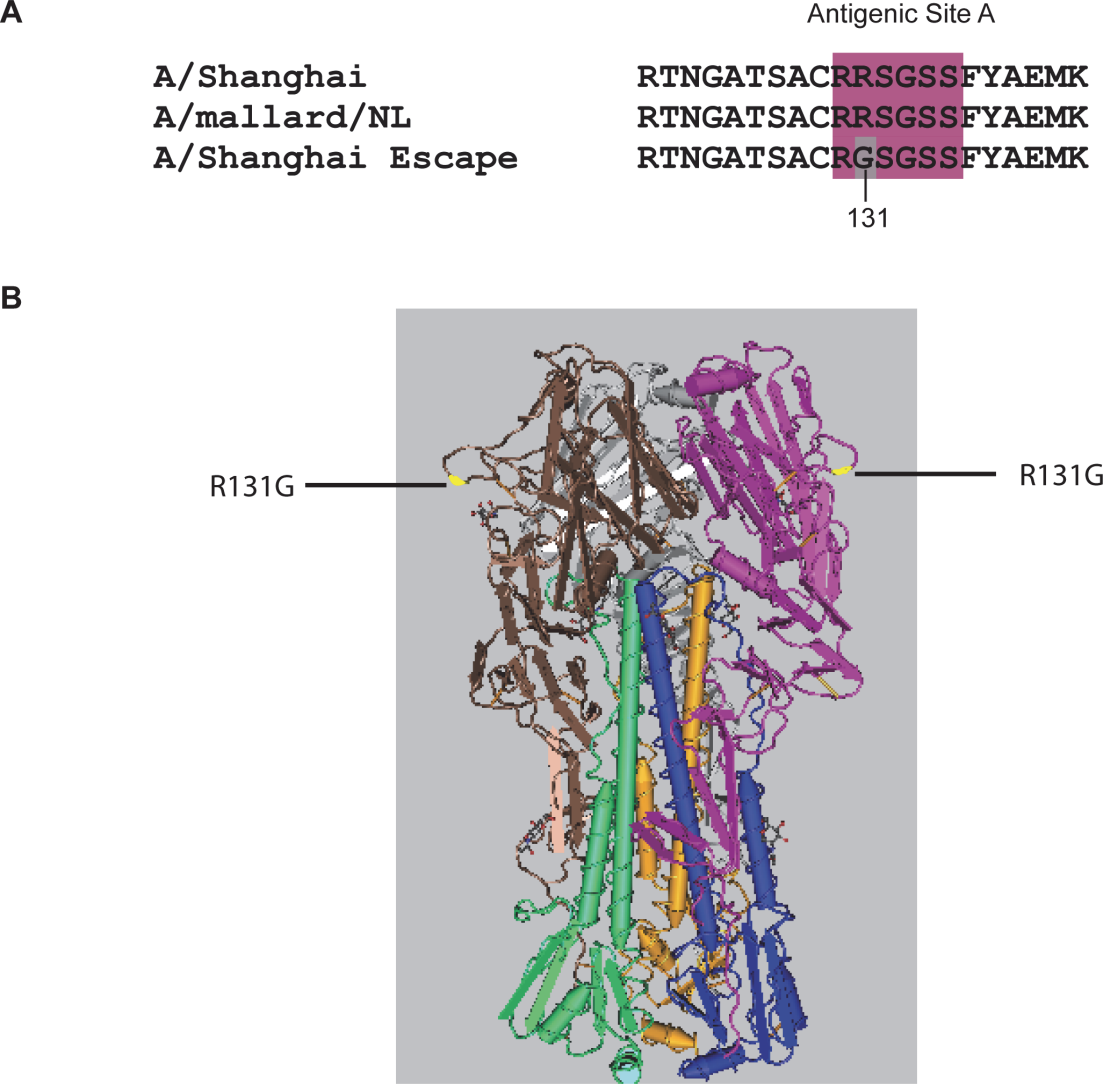
Location of HA amino acid changes in A/Shanghai/2/2013 escape mutants to mAb 5A6, 2C4, and 4A2. **(A)** HA sequence alignment of A/Shanghai/2/2013, escape mutants selected by H7 monoclonal antibodies, and A/mallard/Netherlands/12/2000. The antigenic site A, based on the similar region for H3, is shown in color. The amino acid change identified in the escape mutants is shaded. **(B)** Antigenic structure of the A/Shanghai/2/2013 HA trimer and location of the R131G mutation, highlighted in yellow (PDB ID: 4LN6).

In order to determine the epitope identified by the H7 neutralizing mAbs, escape mutants to A/Shanghai/2/2013 were selected by incubation of A/Shanghai RG32A with each of the neutralizing monoclonal antibodies over a range of concentrations, followed by infection of MDCK cells with the virus-antibody mixtures. Using virus obtained at the highest concentration of mAb, the process was repeated at a 5-fold higher mAb concentration. Following 4 rounds of neutralization, the HA gene of potential escape viruses was sequenced, and the results compared to the sequence of the parent virus. For each mAb, a single arginine to glycine amino acid change was identified at position 131 of the mature HA (position 149 numbered from the first Met of the open-reading frame), confirming that all three neutralizing mAbs recognized the same epitope. Fig. 1 shows the sequence of the escape mutants and the location of the epitope on the HA molecule. The HA epitope recognized by H7 mAbs 5A6, 2C4, and 4A2 overlaps antigenic site A that was defined many years ago for influenza H3 HA [20, 21]. This site is well conserved among H7 hemagglutinins from many influenza strains, including other Eurasian strains such as the earlier H7 vaccine strain containing A/mallard/Netherlands/12/2000 (Fig. 1A), but also HAs from H7 viruses of the North American lineage [22].

**Figure 2:**
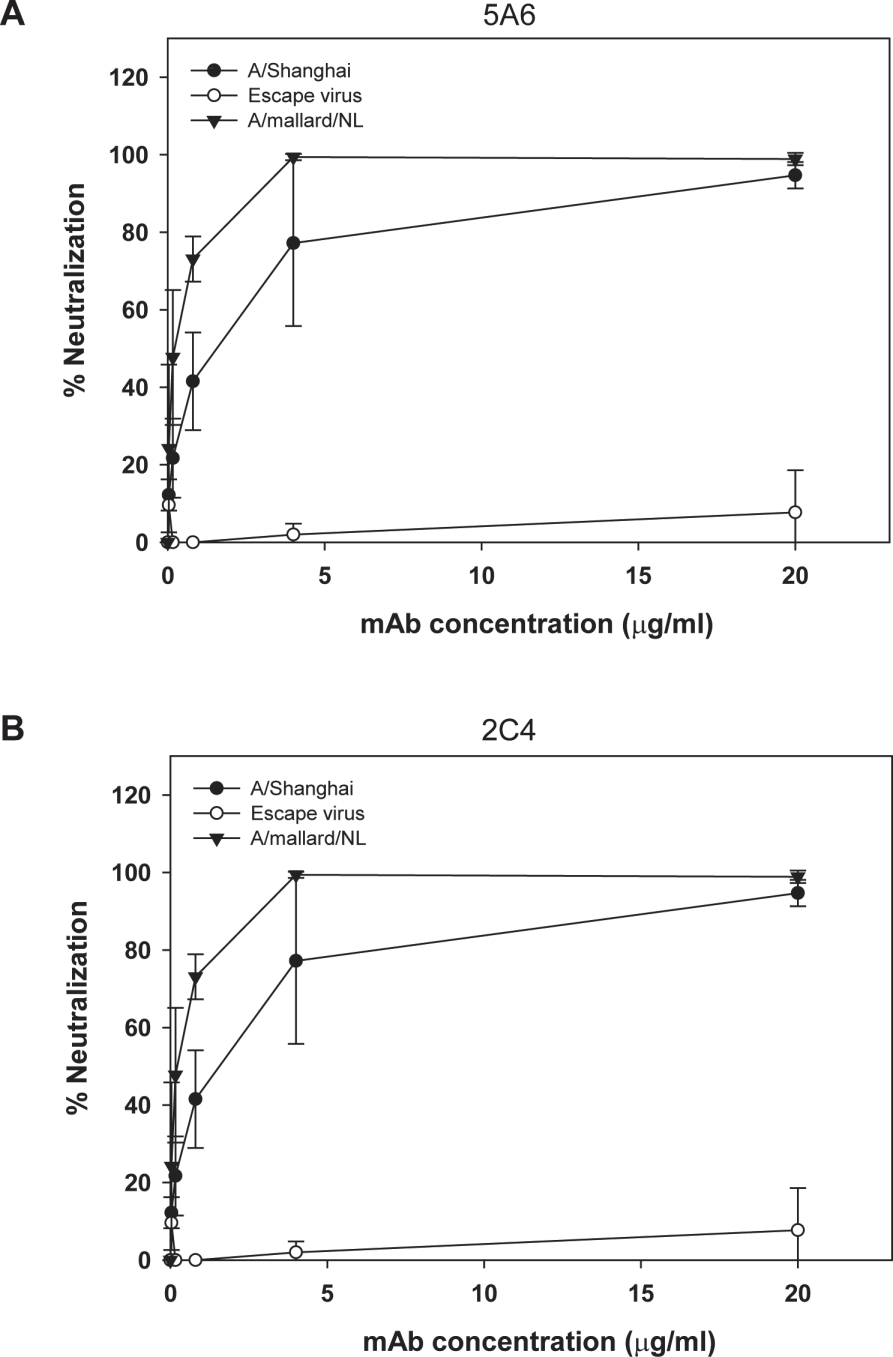
Plaque-reduction neutralization of H7 viruses with H7 mAbs. A/Shanghai/2/2013, A/Shanghai R131G escape virus, and A/mallard/Netherlands/12/2000 H7N3 were incubated with the indicated concentrations of mAb 5A6 **(A)** or 2C4 **(B)** before infection of MDCK cells to determine neutralization. Data are the average (mean) of two replicate experiments.

To further assess the potential importance of an antibody response to the H7 antigenic site A, we measured the neutralizing activity of mAbs 5A6 and 2C4 against A/Shanghai/2/2013 RG32A, the A/Shanghai-derived escape virus containing the R131G mutation, and an H7N3 vaccine virus A/mallard/Netherlands/12/2000 (NIBRG-60) in a plaque reduction neutralization assay (Fig. 2). Each virus was titrated to approximately 250 pfu/ml and incubated with concentrations of each mAb from 20 to 0.032 μg/ml for 1 hour at room temperature before infection of MDCK cells. Percent neutralization was calculated for each mAb concentration versus no mAb controls. Both antibodies had neutralization activity against A/Shanghai/2/2013, with 50% inhibitory concentrations (IC_50_) of approximately 0.95 μg/ml (5A6) and 1.2 μg/ml (2C4), but were unable to neutralize the escape mutant with a R149G mutation even at 20 μg/ml, verifying the escape phenotype conferred by the R131G mutation. Both mAbs readily neutralized the A/mallard/Netherlands/12/2000 virus, which has the same sequence around antigenic site A (Fig. 1A), with IC_50_ of approximately 0.17 μg/ml (5A6) and 0.06 μg/ml (2C4).

### Protection afforded by passive transfer of H7 HA monoclonal antibodies

**Figure 3:**
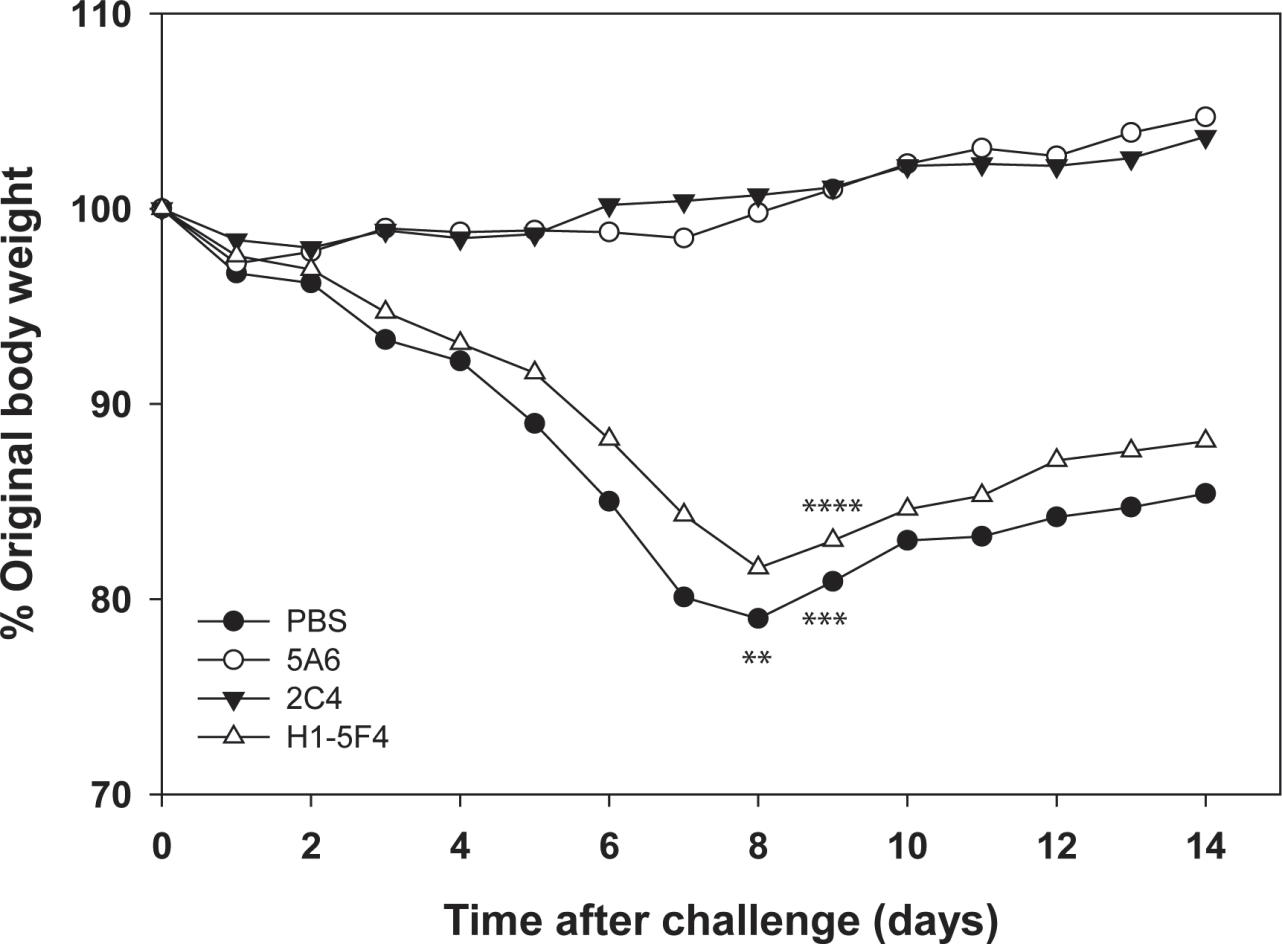
Protection of mice from A/Shanghai/2/2013 challenge. PBS or the indicated mAb (100 μg) were transferred by the intraperitoneal route to mice (groups of 5) approximately 6 hours before being challenged intranasally with A/Shanghai/2/2013. Animals were observed for 2 weeks for weight loss and/or death, and the average percentage change in body weight and deaths (*) in each group are shown. Data are the average (mean) of two replicate experiments.

To assess the protective capacity of the A/Shanghai/2/2013 HA monoclonal antibodies, mice were passively transferred with antibodies prior to challenge with A/Shanghai/2/2013 RG32A. Preliminary experiments determined that this vaccine virus was lethal in mice and established an LD_50_ for subsequent challenge experiments. Antibodies or PBS were injected intraperitoneally into groups of mice approximately 6 hours before intranasal challenge with 1.4 x 10^4^ pfu of A/Shanghai/2/2013 (~1.4 LD_50_). Mice were monitored for 2 weeks for morbidity (body-weight loss) and mortality (Fig. 3). PBS or an unrelated antibody (H1-5F4) failed to protect mice from death (40–50% mortality) or morbidity. Average maximal weight loss in these groups was approximately 20% and occurred 8 days following virus challenge. In contrast, both neutralizing H7 antibodies (5A6 and 2C4) provided protection against challenge. None of the mice in these groups died, and no weight loss was observed in each of 2 replicate experiments. The results indicate that the H7 mAbs with in vitro neutralizing activity are protective in vivo in this mouse challenge model.

## Discussion

At the time of the emergence of deadly H7N9 viruses in China in 2013, only a limited number of candidate vaccine strains had been prepared to influenza H7 subtypes. The available data indicated that the antibody response to HA in experimental vaccines was relatively poor compared to seasonal influenza strains. More recently, a study examining the antibody responses to H7N9 infection seemed to suggest that the natural immune response to H7 HA was weak [23]. Clinical trials undertaken with H7N9 vaccines prepared in response to the H7N9 emergence were encouraging, but the need for adjuvants and 2 doses of vaccine seemed to confirm the expectations of relatively poor HA immunogenicity. Compounding the problem of poor immunogenicity, very little is known about what constitutes a protective immune response to the H7 subtype of influenza.

The majority of neutralizing antibodies to influenza, elicited by infection or vaccination, recognize the globular head of HA. Five distinct antigenic sites on the HA have been defined and characterized, designated as either antigenic sites A-E using influenza H3 [20, 21] or Sa, Sb, Ca1, Ca2, and Cb using influenza H1 [24, 25]. As a consequence of amino acid sequence variability in these sites, the antibody response to infection or vaccination is highly strain-specific. Although the major antigenic sites for other influenza HA subtypes such as H7 are less well understood, the conserved structure of the HA receptor binding site in the globular head suggests that the previously defined antigenic sites will be similar, particularly for HA subtypes within phylogenetic Group 1 (e.g., H1, H5) and Group 2 (e.g., H3, H7). Indeed, a recent alignment of H7 hemagglutinin sequences in comparison with the structurally similar H3 HA is revealing [22]. Putative antigenic sites A-E were identified in the H7 HA, although with extensive variability relative to the analogous antigenic sites in H3. Interestingly, the amino acid sequences of the compared H7 HAs were relatively well conserved in this analysis. Further, sera from animals immunized with a North American lineage H7 HA was cross-reactive to a broad range of H7 viruses, suggesting the importance of conserved antigenic sites in the H7 HA.

Identifying major antigenic and protective epitopes of the H7 hemagglutinin will be important for understanding vaccine responses as H7 vaccine development and evaluation continues. Toward this goal, we generated a panel of murine monoclonal antibodies to the HA of A/Shanghai/2/2013 and identified mAbs that were neutralizing. All of the neutralizing antibodies obtained in this initial study were directed to the same antigenic site, a region analogous to the antigenic site A of influenza H3 hemagglutinin. The immunogen used for immunization was a full-length mammalian expressed HA, presented as a virus-like particle. We do not know at this time whether the failure to isolate neutralizing antibodies to other HA epitopes is meaningful or just the chance result in a relatively small number of hybridoma clones, but additional studies to isolate and screen more mAbs are underway.

A mouse model of infection was used to assess protection afforded by these site A neutralizing antibodies. The challenge virus used in these studies was a candidate vaccine virus that resulted in both morbidity and lethality at doses easily achievable without virus concentration. In addition, use of a candidate vaccine virus for challenge only required BSL2 level containment. Importantly, the two individual neutralizing H7 mAbs tested in this model provided protection against challenge compared to control mAbs or mock-treatment, suggesting that the identified antigenic site is also a potentially important protective epitope. Additional studies, using a wider range of antibody concentrations and stronger challenge doses, would be needed in order to assess the relative strength of protection afforded by these mAbs in animal models of protection. Such studies would ideally be performed in conjunction with additional neutralizing mAbs targeting other H7 epitopes to better define the role of specific antigenic epitopes in a protective antibody response. Finally, understanding how antibodies elicited to antigenic site A and other antigenic sites in the H7 HA contribute to protection in humans will require a detailed study of antibody responses to vaccination, correlated with efficacy and effectiveness studies.

Antigenic site A is relatively well conserved among H7 virus isolates, including older vaccine strains such as the A/mallard/Netherlands/12/2000 strain used in a previous clinical trial. Plaque reduction neutralization assays using this virus confirmed the cross-reactivity of the neutralizing mAbs isolated to the A/Shanghai/2/2013 HA, as would be predicted from the analysis of amino acid sequences of the respective HAs. In fact, the neutralizing activity of the mAbs against A/mallard/Netherlands/12/2000 appeared to be even stronger than against the A/Shanghai/2/2013 virus strain (Fig. 2) by 5- to 19-fold, 5A6 and 2C4, respectively. The 95% confidence limits of the IC_50_ calculated for 2C4 against A/mallard/Netherlands/12/2000 and A/Shanghai/2/2013 overlap, so the significance of the measured differences in neutralizing activity are not clear at this time. Nevertheless, differences in the overall structures of A/mallard/Netherlands/12/2000 and A/Shanghai/2/2013 HA that could affect mAb binding to an identical antigenic site A in each HA cannot be ruled out. Although we have not yet set-up an animal challenge model with the A/mallard/Netherlands/12/2000 virus strain, comparison of the plaque-reduction neutralization results would suggest that the A/Shanghai/2/2013 mAbs would be protective against A/mallard/Netherlands/12/2000. Additional neutralization and protection studies would be needed to confirm the cross-reactivity of the A/Shanghai/2/2013 mAbs with other H7 strains as predicted by sequence comparisons.

In summary, the results presented here show that an H7 antigenic site A, predicted by comparison and alignment with the structurally similar H3 HA, may be important for a protective response in vaccination. The conservation of this site among H7 isolates, including strains of both Eurasian and North American lineage, suggests potential benefit of cross-reactive antibody responses to vaccination with H7 candidate vaccines and emphasizes the importance of further studies to identify protective epitopes on the H7 hemagglutinin. A better understanding of H7 protective epitopes and the specific antibody response to candidate vaccine viruses would be extremely important for vaccine policy decisions in the event of a pandemic.
